# *Aspergillus texensis*: A Novel Aflatoxin Producer with S Morphology from the United States

**DOI:** 10.3390/toxins10120513

**Published:** 2018-12-03

**Authors:** Pummi Singh, Marc J. Orbach, Peter J. Cotty

**Affiliations:** 1 School of Plant Sciences, The University of Arizona, Tucson, AZ 85721, USA; orbachmj@email.arizona.edu (M.J.O.); Peter.Cotty@ARS.USDA.GOV (P.J.C.); 2 USDA-ARS, 416 W Congress St, First Floor, Tucson, AZ 85701, USA

**Keywords:** Aflatoxins, *Aspergillus texensis*, S morphology, Molecular phylogenetics

## Abstract

Aflatoxins are carcinogenic metabolites produced primarily by fungi within *Aspergillus* section *Flavi*. These fungi infect a wide range of crops in warm regions. Molecular phylogenetic analyses of fungi with S morphology (average sclerotium size < 400 µm) within section *Flavi* collected from across the United States (US) resulted in the discovery of a novel aflatoxin-producing species, *Aspergillus texensis*. *Aspergillus texensis* was isolated from maize grown in Arkansas, Louisiana, and Texas, and from soils cropped to maize in Texas. *Aspergillus texensis* produces sparse conidia and abundant sclerotia on various culture media, and on maize. Physiological studies have revealed optimal growth on culture media at 35 °C. All isolates of *A. texensis* produced B and G aflatoxins, cyclopiazonic acid and aspergillic acid. *Aspergillus texensis* and *A. flavus* S strain morphotypes produced similar concentrations of total aflatoxins on maize (*p* > 0.05). Phylogenetic analyses of aflatoxin-producers based on partial gene sequences of the β-tubulin (0.9 kb), calmodulin (1.2 kb), and nitrate reductase (2.1 kb) genes placed *A. texensis* in a highly supported monophyletic clade closely related to *A. minisclerotigenes* and a previously reported unnamed lineage designated Lethal Aflatoxicosis Fungus.

## 1. Introduction

Aflatoxins (AF) are extremely potent naturally-occurring hepatocarcinogenic mycotoxins produced by several members of the genus *Aspergillus* on important commodities such as maize, groundnut, tree nuts, spices, and cottonseed [1,2,3,4,5]. Naturally occurring aflatoxin-producers contaminate food and feed with four major aflatoxins, i.e., aflatoxins B_1_, B_2_, G_1_ and G_2_. Bio-transformation of aflatoxins from consumption of contaminated food results in formation of aflatoxins M_1_ and M_2_, which are secreted into milk [6,7]. Although certain species within *Aspergillus* section *Ochraceorosei* and *Aspergillus* section *Nidulantes* also produce aflatoxins [8,9], the most economically important aflatoxin-producers belong to *Aspergillus* section *Flavi* [10,11,12]. *Aspergillus flavus, A. parasiticus, A. aflatoxiformans* from West Africa, and an unnamed lineage referred to as the Lethal Aflatoxicosis Fungus (LAF) from Kenya are particularly notorious members of *Aspergillus* section *Flavi* responsible for contamination of crops with high levels of aflatoxins [3,13,14,15,16,17]. These fungi colonize a wide range of host crops resulting in dangerous concentrations of aflatoxins under conducive environmental conditions (high temperature, i.e., >28 °C, humidity and plant stress) [18]. 

Aflatoxins are both health and economic threats. Aflatoxin B_1_ has been categorized as a Group 1 human carcinogen by IARC [19]. In developing nations, aflatoxin regulations largely remain unenforced and crops are consumed without monitoring, resulting in frequent exposure of humans and animals [20,21]. Chronic dietary exposure to sub-lethal concentrations can cause immune suppression [22], impaired growth [23,24], and liver cancer [25,26]. Ingestion of high concentrations of aflatoxins may result in liver necrosis followed by rapid death [20,21]. Acute cases of aflatoxin poisoning have resulted in human deaths in Kenya and Tanzania [27,28,29]. Aflatoxins are a severe economic burden in the developed world where regulations are stringently enforced leading to heavy economic losses incurred by growers [30]. These regulatory enforcements result in rejection of contaminated food/feed followed by loss of markets and expense to the exporter. Since aflatoxins remain a global concern, it becomes crucial to identify and characterize aflatoxin-producing species in order to design management strategies for aflatoxin contamination of crops. 

*Aspergillus flavus* and *A. parasiticus* are the most commonly implicated causal agents of aflatoxin contamination of crops [31]. The filamentous fungus *A. flavus* produces only B aflatoxins and has two morphotypes. The L strain morphotype is characterized by the production of copious conidia, few large sclerotia (>400 µm), and variable concentrations of aflatoxins, such that many L strain morphotype fungi are atoxigenic (do not produce aflatoxins); the S strain morphotype produces large quantities of small sclerotia (<400 µm) and sparse conidia [32]. The *A. flavus* S strain morphotype consistently produces high concentrations of aflatoxins. *Aspergillus parasiticus* produces both B and G aflatoxins. The *A. flavus* S strain morphotype produces only B aflatoxins and is the more commonly occurring S morphology fungus in North America [2,32,33]. However, several highly aflatoxigenic S morphology fungi belonging to genetically distinct taxa, i.e., *A. minisclerotigenes*, *A. aflatoxiformans*, *A. cerealis* (previously *A. korhogoensis*), and LAF, are known from Sub-Saharan Africa [4,14,15,17,34]. During phylogenetic analysis of fungi with S morphology belonging to *Aspergillus* section *Flavi* isolated from soils and maize collected from across the US, we discovered fungal isolates that produced both B and G aflatoxins, but were morphologically indistinguishable from the *A. flavus* S strain morphotype. Phylogenetic reconstruction using multi locus gene sequences with previously described members of *Aspergillus* section *Flavi* indicated that these B and G aflatoxin producers represent a novel, undescribed species.

*Aspergillus* section *Flavi* contains several phylogenetically distinct species with S morphology characterized by production of small sclerotia (<400 µm) [16,17,32]. Description of species within section *Flavi* solely based on phenotypic characteristics can be erroneous due to overlapping character states [35,36]. The current study used a polyphasic approach to compare newly discovered species to those previously described. Phenotypic description included macro- and micromorphology, growth at different temperatures, and production of aflatoxins, aspergillic acid, and cyclopiazonic acid. Phylogenetic reconstructions based on multiple unlinked loci were utilized to determine relationships of the novel species to those previously described with S morphology.

## 2. Results

### 2.1. Molecular Analyses and Phylogenetics

**Figure 1:**
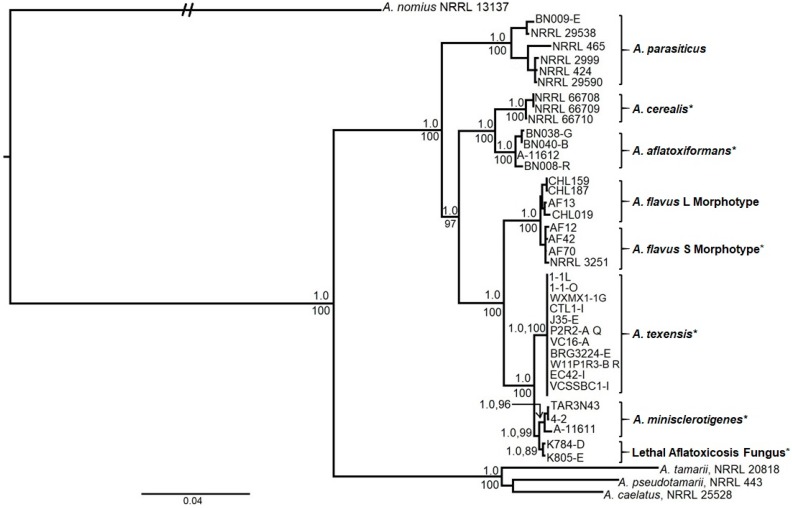
Mid-point rooted Bayesian phylogeny of *A. texensis* and closely related S morphology fungi with several additional species within section *Flavi* for reference. Phylogeny is based on concatenated *benA* (0.9 kb), *cmdA* (1.2 kb), and *niaD* (2.1 kb) genes of *Aspergillus* section *Flavi*. Values above nodes or before commas are Bayesian posterior probabilities and values below nodes or after commas are bootstrap values from 500 replicates. *Aspergillus nomius* NRRL 13137 was used as the outgroup. * Aflatoxin producers with S morphology.

Homologous DNA sequences of reference isolates of described species within section *Flavi* with S morphology (e.g., *A. flavus* S strain morphotype, *A. minisclerotigenes, A. cerealis, A. aflatoxiformans*), representatives of the fungi associated with lethal aflatoxicosis in Kenya (LAF), and other aflatoxin-producers (*A. nomius, A. pseudotamarii, A. parasiticus*) were aligned with sequences of S morphology fungi recovered from the US (Table 1). Phylogenetic reconstruction using Bayesian Inference (BI) and Maximum Likelihood (ML) analyses from partial gene sequences of β-tubulin (*benA*, 0.9 kb, chromosome 6), calmodulin (*cmdA*, 1.2 kb, chromosome 2), and nitrate reductase (*niaD*, 2.1 kb, chromosome 4) genes for individual and concatenated sequences yielded similar topologies. The new species, named *Aspergillus texensis*, occupied a highly supported monophyletic clade in concatenated *benA*, *cmdA,* and *niaD* gene phylogenies (Figure 1), and was sister to a clade containing *A. minisclerotigenes* and LAF fungi. Phylogenetic reconstruction based individually upon *cmdA* and *niaD* sequences showed that each of these genes was sufficient to resolve *A. texensis* into a monophyletic clade with high Bayesian posterior probability and bootstrap support (Figures S1 and S2). The *A. flavus* S strain morphotype, which is a frequently reported S morphology fungus in the US, is phylogenetically distinct from *A. texensis* (Figure 1). Additionally, molecular analysis of the *norB-cypA* region did not detect any deletions in the *cypA* gene of *A. texensis,* whereas the *A. flavus* S strain morphotype isolates contained either a 0.9 or 1.5 kb deletion in this region of the aflatoxin biosynthesis cluster required for G aflatoxin production. 

All isolates of *A. texensis*, regardless of state of origin, contained only the *MAT1-1* idiomorph at the mating-type locus, suggesting that *A. texensis* is heterothallic. Each isolate amplified only a single amplicon of approximately 390 bp in the MAT locus PCR assay. This is characteristic of the *MAT1-1* idiomorph. The *MAT1-2* idiomorph that is highly conserved in *Aspergillus* section *Flavi* should produce a 270 bp amplicon, but was not detected in any *A. texensis* isolate.

### 2.2. Aspergillic Acid

**Figure 2:**
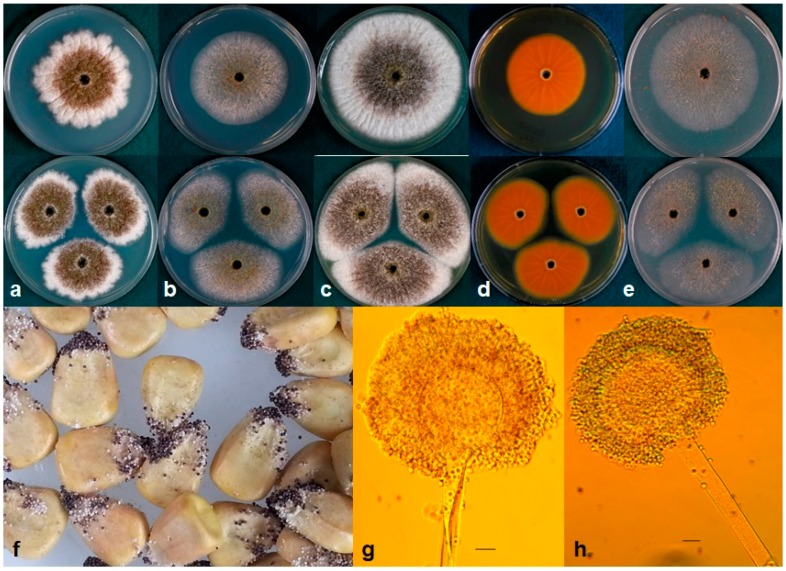
(**a**–**e**) Colonies of *A. texensis* grown at 25 °C for 7 d on (**a**) CZ, (**b**) CYA, (**c**) Malt agar, (**d**) reverse on AFPA, and (**e**) V8 agar. (**f**) *A. texensis* on maize grown at 31 °C for 7 d; (**g**–**h**) Conidiophores. Bars—10 µm.

All isolates of *A. texensis* produced aspergillic acid, which was detected by the bright-orange reaction on the reverse side of AFPA medium (Figure 2; Table 2), similar to *A. flavus* and *A. minisclerotigenes*. Colony texture of *A. texensis* on AFPA after 7 d at 25, 30 and 35 °C was floccose with abundant white mycelia and immature white sclerotia.

### 2.3. Cyclopiazonic Acid

*Aspergillus texensis* produced cyclopiazonic acid (CPA) (Table 2); CPA concentrations ranged from 7.5–26.6 µg/g with a mean of 17.4 µg/g. Similarly, all S strain morphotype isolates of *A. flavus* (n = 3) tested in the current study produced CPA (Range: 16.0–30.6 µg/g; Mean = 25.0 µg/g).

### 2.4. Aflatoxins

*Aspergillus texensis* produced B_1_, B_2_, G_1_, and G_2_ aflatoxins in maize. Aflatoxin concentrations ranged from 11–71 µg/g AFB_1_, 0.6–2.6 µg/g AFB_2_, 66–225 µg/g AFG_1_, and 2.0–7.7 µg/g AFG_2_. The S strain morphotype of *A. flavus*, which is the most commonly reported S morphology fungus in the US, produces only B aflatoxins. In an experiment comparing total aflatoxin production by genetically distinct S morphology fungi, mean aflatoxin concentrations produced by *A. texensis* and *A. flavus* S morphotype fungi were similar at 31 °C (Table 3; *p* > 0.05). *Aspergillus texensis* produced at least two-fold more G aflatoxins than B aflatoxins (Table 3). Fungi with S morphology from the African continent, including isolates of *A. aflatoxiformans* and LAF, produced the highest concentrations of total aflatoxins in maize, while *A. minisclerotigenes* produced the lowest quantities. However, all fungi with S morphology evaluated in the current study overall produced lethal concentrations of total aflatoxins in maize at 31 °C (Range: 33–568 µg/g).

### 2.5. Taxonomy

***Aspergillus texensis*** P.Singh, M.J.Orbach, and P.J.Cotty **sp. nov.** MycoBank MB828668. Figure 2. 

*Etymology*: The species epithet *texensis* is Latin for “from Texas”, where the first isolates of the new species were collected. 

*Diagnosis*: *Aspergillus texensis* is closely related to *A. minisclerotigenes* and the unnamed lineage LAF. *Aspergillus minisclerotigenes* grows faster than *A. texensis* on Czapek agar at 20 °C and 37 °C, and on V8 agar at 37 °C; however, *A. texensis* grows faster on V8 agar at 40 °C (Table 4). The unnamed lineage LAF lacks the ability to produce G aflatoxins unlike *A. texensis,* which produces both B and G aflatoxins (Table 3).

*Typus:* United States of America, Texas, Waxahachi, soil cropped to maize (*Zea mays*), collected by P.J.Cotty (holotype NRRL 66855, ex-type: WXMXP1R3-B R = A2292).

*Colony* characteristics: *Aspergillus texensis* colonies attained an average diameter of 53 mm (51–58 mm) at 25 °C, 55 mm (53–59 mm) at 37 °C, and 7 mm (4–9 mm) at 42 °C on Czapek agar after 7 d. Colony diameters were greater on Czapek agar with 2% NaCl and Czapek agar with yeast extract (CYA) media at these temperatures (Table 4). Maximum radial growth of *A. texensis* occurred at 35 °C on all media. *Aspergillus texensis* did not germinate at 5 °C or 10 °C. Colony surface on Malt agar was floccose with dominant white mycelia, velvety on CYA and V8 (Figure 2). Colony reverse buff on CZ, CYA and Malt.

*Micromorphology:* Abundant production of dark black sclerotia (150–300 µm) on the agar surface was observed on Czapek, CYA, Malt, and V8 agar (Figure 2). Fungal isolates produced sparse yellow-green conidia on all media tested; conidia were circular and smooth walled (3-6 µm diameter). Vesicle globose, 30–60 µm in diameter. Conidiophores with stippled stipes, hyaline, 400–800 × 10–14 µm, phialides 6–11 × 3–5. *Aspergillus texensis* produced copious quantities of sclerotia but fewer conidia on maize after incubation at 31 °C for 7 d (Figure 2). 

The type and other representative isolates of *A. texensis* have been submitted to the ARS Culture Collection (NRRL) (Peoria, IL, USA) and the Fungal Genetics Stock Center, Manhattan, KS (Table 1).

## 3. Discussion

Most aflatoxin producing fungi belong to *Aspergillus* section *Flavi* with several members recognized as economically important agents of aflatoxin contamination of crops [13]. Although many species within section *Flavi* produce aflatoxins, fungi that have repeatedly been recovered at high frequencies from crops and agricultural soils, such as *A. flavus* L and S strain morphotypes, *A. parasiticus, A. minisclerotigenes, A. aflatoxiformans* from West Africa, and the fungi associated with lethal aflatoxicoses in Kenya (LAF), are the primary concern as causal agents of aflatoxin contamination of crops [1,12,14,15,16]. Morphology (sclerotia, conidia, colony characteristics) and physiology (growth rates at different temperatures), and mycotoxin production have been conventional tools in identification and characterization of species within section *Flavi* [41,46]. However, in the past decade, fungi within section *Flavi* with overlapping morphological and physiological characteristics have been assigned to genetically distinct taxa based on DNA sequences [16,17,34,42]. For instance, the *A. flavus* S Strain morphotype and LAF fungi produce small sclerotia (<400 µm) and only B aflatoxins; however, multi locus gene genealogies, and differences in deletions in the *norB-cypA* region of the aflatoxin biosynthesis gene cluster, clearly show that *A. flavus* and LAF are distinct species [16,44]. Likewise, *A. minisclerotigenes*, *A. aflatoxiformans*, and *A. cerealis* show high morphological similarity (numerous small sclerotia), and produce B and G aflatoxins. DNA sequence data from multiple unlinked loci strongly support placement of each of these into a distinct monophyletic taxon [17,34,42]. The utilization of a polyphasic approach, which combines morphology, physiology, and DNA-based analyses for recognition of novel species, provides a powerful tool for researchers to approach cryptic diversity within *Aspergillus* section *Flavi.* However, multi-locus DNA sequence-based phylogenetics are frequently sufficient for delineation of new taxa [17,47,48]. 

*Aspergillus texensis* forms a highly supported distinct terminal group in phylogenies of sequence data from three unlinked loci (*benA, cmdA, niaD*). The branch containing *A. texensis* is congruent, and the same isolates occur as a terminal group in trees constructed from individual or concatenated genes with strong statistical support, both by Bayesian posterior probability and bootstrap analyses (Figure 1; Figures S1 and S2). In light of the aforementioned results, *A. texensis* fulfills requirements of the phylogenetic species concept, which limits species boundaries to a monophyletic group within which a pattern of ancestry and descent exists [36,47,49]. This, along with physiological data and types of secondary metabolites produced, distinguishes *A. texensis* as a new species. 

DNA sequences resulting from the portion of the β-tubulin gene amplified using primers from [50] were highly conserved among closely-related aflatoxin producing species. The current study therefore incorporated partial 5′ untranslated region (UTR) of β-tubulin using primer pair Bt3a-3b (Table 5) to include more variable characters to seek better resolution. However, 0.9 kb of individual β-tubulin sequences (112 5′ UTR positions, 320 exon positions and 453 intron positions) still did not contain enough variable characters to resolve multiple aflatoxin producing species. Nevertheless, individually, calmodulin and nitrate reductase sequences could clearly separate previously reported aflatoxin-producers and *A. texensis* in both Bayesian and Maximum Likelihood topologies with high Bayesian posterior probabilities and Bootstrap support (Figures S1 and S2). The coding sequence of β-tubulin encodes for proteins, which along with alpha tubulin, polymerize into microtubules, cellular structures that are crucial in multiple cellular processes. Owing to this highly conserved function, DNA sequences of β-tubulin may not be sufficient to reveal the cryptic diversity among more closely related species within *Aspergillus* section *Flavi,* while calmodulin and nitrate reductase could be superior choices to identify novel species within section *Flavi.* This is in agreement with previous studies describing novel species within section *Flavi* that have often reported phylogenies based on concatenated gene sequences including β-tubulin, calmodulin, RNA polymerase, internal transcribed spacers (ITS), etc. with high support for the clade representing a new species [34,36,48]. However, gene trees based on individual sequences of RNA polymerase, ITS, and β-tubulin either did not resolve members of section *Flavi* or did so with low branch support [36,48]. On the other hand, the calmodulin gene provides a useful tool to identify and resolve closely-related aflatoxin producers of section *Flavi* [17,35,36,48]. The nitrate reductase gene, which encodes for an enzyme essential for nitrate assimilation in fungi, is also a powerful tool to identify and characterize species within section *Flavi,* as demonstrated in the current study, and by [4,12].

The fungal isolates representing *A. texensis* sp. nov. produce abundant quantities of small sclerotia (<400 µm diameter) on several growth media, and on maize kernels (Figure 2). Conidia are smooth walled and appear light green to yellowish green, similar to the S strain morphotype of *A. flavus*. Although microscopic characters and growth at different temperatures in various media largely overlap (Table 4), *A. texensis* is characterized by production of both B and G aflatoxins, unlike the *A. flavus* S strain morphotype, which produces only B aflatoxins. This is due to either a 0.9 or 1.5 kb deletion in the *norB*-*cypA* region of the aflatoxin biosynthesis gene cluster in *A. flavus,* which has resulted in loss of G aflatoxin production by this species [51]; however, *A. texensis* has an intact *norB*-*cypA* region. Total aflatoxin production on maize by *A. flavus* S strain morphotype did not differ from that of *A. texensis* (Table 3; *p* > 0.05); however, *A. texensis* produced significantly lower concentrations of B aflatoxins (Table 3; *p* < 0.05). Additionally, phylogenetic reconstruction based on 4.1 kb sequence data from three unlinked genes (*benA*, *cmdA* and *niaD*) clearly demonstrate that *A. texensis* is a genetically distinct, distant relative of *A. flavus*. 

*Aspergillus texensis* is closely related to *A. minisclerotigenes* (first described from Argentinian groundnuts) [42] and LAF (the lineage with S morphology responsible for severe aflatoxin contamination that led to hundreds of deaths in Kenya) [16,44] (Figure 1). Both *A. minisclerotigenes* and LAF have been reported in very low frequencies in the US (one isolate of *A. minisclerotigenes* and three isolates of LAF) [16,42]. However, higher incidences of *A. minisclerotigenes* are known from groundnuts in Argentina [42], almonds and maize in Portugal [48], maize in Eastern and Central Africa [4], and chilies in Nigeria [12]. Similarly, LAF has been recovered in high frequencies from maize in Kenya [16]. Production of G aflatoxins by *A. texensis* differentiates it from LAF, which produces only B aflatoxins due to a characteristic 2.2 kb deletion in the *norB*-*cypA* region of the aflatoxin biosynthesis gene cluster [16]. *Aspergillus texensis* is distinguished in growth from *A. minisclerotigenes* on Czapek agar at 20 °C and 37 °C, and on V8 agar at 37 °C and 40 °C (Table 4). Furthermore, *A. texensis* produces higher concentrations of aflatoxins than *A. minisclerotigenes* on maize (Table 3; *p* < 0.05). Also, phylogenetic analyses of DNA sequence data strongly support the delineation of these two species (Figure 1, Figures S1 and S2). 

Fungal isolates of most species within section *Flavi* contain either *MAT1-1* or *MAT1-2* idiomorphs at the mating type locus, and are therefore heterothallic [34]. All isolates of *A. texensis* examined to date have the *MAT1-1* idiomorph suggesting clonal evolution in the absence of meiotic recombination, which within heterothallic species requires the presence of *MAT1-2* idiomorph strains for sexual reproduction. However, further sampling is needed to determine whether isolates of *A. texensis* with the *MAT1-2* idiomorph are present, as only 11 isolates have been collected to date. 

Aflatoxigenic fungi frequently reported across the US include *A. flavus* L strain morphotype (isolates can be toxigenic or atoxigenic), *A. flavus* S strain morphotype, and *A. parasiticus* [52,53,54,55]. Accurate identification and characterization of aflatoxin producing fungi allows clarification of the etiology of crop contamination and development of procedures for aflatoxin mitigation. *Aspergillus texensis* is known from 11 isolates currently, and was recovered from soils cropped to maize, and maize grain produced in Arkansas, Louisiana, and Texas. Aflatoxin contamination of crops is severe in these regions [56,57,58,59]. The majority of *A. texensis* isolates (82%) discovered in the current study are from Texas, where contamination is a perennial issue. The highly toxic *A. flavus* S strain morphotype has been reported in high frequencies from hot and dry regions of Texas, and increased proportions of these fungi have been associated with increased soil temperature [33,60]. The morphology of *Aspergillus texensis* is highly similar to that of the S strain morphotype of *A. flavus* with numerous small (<400 µm) sclerotia. Furthermore, both *A. texensis* and the *A. flavus* S strain morphotype produce high and comparable concentrations of aflatoxins in maize. These data, along with the aforementioned observations, suggest that co-occurrence of these S morphology aflatoxin producers in the US may lead to crop contamination with high aflatoxin concentrations. The current study also indicates that G aflatoxins in maize are not solely attributable to *A. parasiticus*.

## 4. Materials and Methods

### 4.1. Fungal Isolates and Morphology

Eleven fungal isolates were chosen from a survey of S morphology fungi, collected from across the US, based on their ability to produce both B and G aflatoxins. These isolates were recovered from soil and maize samples at the USDA-ARS laboratory in Tucson, Arizona. Fungal isolates with known phylogenetic placement were obtained from the ARS Culture Collection, Peoria, IL, USA (NRRL in Table 1), the American Type Culture Collection, Manassas, USA (ATCC in Table 1), the Fungal Genetics Stock Center, Manhattan, KS, USA (A in Table 1), or the USDA-ARS Laboratory Culture Collection in Tucson, Arizona (Table 1).

In order to compare morphological features and growth, fungal isolates were grown for 7 d in the dark at 5 °C, 10 °C, 15 °C, 20 °C, 25 °C, 30 °C, 35 °C, 37 °C, 40 °C, and 42 °C using center point inoculation on Czapek agar (20 g Bacto agar, 30 g sucrose, 3 g NaNO_3_, 0.5 g KH_2_PO_4_, 0.5 g K_2_HPO_4_, 0.5 g MgSO_4_ * 7H_2_O, 0.5 g KCl, and 1 mL micronutrients per liter of deionized distilled water, pH = 6.0), Czapek with 2% NaCl, Czapek yeast extract agar (CYA) (Czapek agar with 0.5% yeast extract), Malt agar and V8 agar (5% V8 juice and 2% agar, pH = 6.0). Micronutrients were prepared according to [61]. Colony diameters were recorded (four replicates per isolate) at each temperature. For micromorphological observations, 3 d old cultures grown on Czapek agar were viewed and captured with a differential interference contrast microscope (Model BH-2, Olympus, Shinjuku, Tokyo, Japan) equipped with an OMAX 5.0MP USB2.0 digital camera and the software package ToupView (v 3.7, ToupTek Photonics, Hangzhou, China, 2014). 

### 4.2. Production of Aspergillic Acid, Aflatoxins, and Cyclopiazonic Acid

Aspergillic acid production was analyzed by inoculating fungal isolates onto *Aspergillus flavus* and *parasiticus* agar (AFPA) [62], which was incubated for 7 d in the dark at 25 °C, 30 °C, and 35 °C. Isolates were replicated four times at each temperature. 

Aflatoxins were analyzed and quantified for fungi with S morphology belonging to five phylogenetically distinct taxa. Each taxon was represented by three isolates. Conidial suspensions were inoculated onto 10 g autoclaved maize (Pioneer hybrid N82VGT), as previously described [63]. Briefly, single spored isolates were seeded at the center of V8 agar plates and incubated for 7 d at 31°C. Conidia were swabbed from plates with sterile cotton swabs into sterile distilled water (10 mL). The quantity of conidia was measured using a turbidity meter (Turbidimeter, Orbeco Analytical Systems) and maize was inoculated with 10^6^ conidia/mL of each fungal isolate. Water content of maize was adjusted to 30%. Maize cultures were incubated at 31 °C for 7 d in the dark to allow fungal growth and aflatoxin formation. The experiment was terminated by addition of 50 mL 85% acetone and crop cultures were ground to homogeneity in a laboratory grade Waring Blender (seven-speed laboratory blender, Waring Laboratory, Torrington, CT, USA) at full speed for 30 s. The culture filtrate was spotted directly onto thin-layer chromatography (TLC) plates (Silica gel 60, EMD, Darmstadt, Germany) adjacent to aflatoxin standards (Aflatoxin Mix Kit-M, Supelco, Bellefonte, PA, USA) containing a mixture of known concentrations of aflatoxins B_1_, B_2_, G_1_, and G_2_. Plates were developed in ethyl ether-methanol-water (96:3:1), air-dried, and aflatoxins were visualized under 365-nm UV light. Total aflatoxins were quantified directly on TLC plates with a scanning densitometer (TLC Scanner 3, Camag Scientific Inc., Wilmington, NC, USA). Treatments were replicated four times and each experiment was performed twice.

Cyclopiazonic acid analyses were performed according to [64]. Conidial suspensions were inoculated onto 20 g of autoclaved maize using the method described above. For CPA analyses, inoculated maize was incubated for 7 d at 31 °C in the dark, and the experiment was terminated by addition of 100 mL of extraction solvent (20% methanol, 80% chloroform and 0.2% of 85% phosphoric acid). Maize-fungal cultures were ground at full speed to homogeneity in a laboratory grade Waring Blender. The slurry was transferred to polypropylene containers and shaken for 60 min at 200 rpm on a rotary shaker (HS501digital, IKA Works Inc., Wilmington, NC, USA). Extracts were filtered through Whatman No. 4 filter paper into 100 mL cylinders and the volume of extract was recorded. Each extract was transferred into a separatory funnel followed by addition of 100 mL of 0.5 N NaHCO_3_ with 3% (*w*/*v*) NaCl and gently shaken. The mixture was allowed to settle for 30 min and the bottom layer was discarded. Seven mL of concentrated HCl was added dropwise to each sample and gently shaken for 30 s. Once the bubbling subsided, samples were extracted twice with 25 mL of chloroform. Extracts were combined, dried, and re-suspended in 4 mL of chloroform. Extracts were spotted directly on TLC plates alongside a CPA standard of known concentration. Plates were dried at 50 °C for 15 min and then cooled for 1 min. Each plate was then dipped in Ehrlich’s reagent, quickly removed and air dried. Appearance of purple bands alongside the CPA standard indicated that samples were positive for CPA. *Aspergillus flavus* L strain isolate AF36, which is a registered biological control in the US, was used as the positive control [65]. CPA was quantified on TLC plates by scanning fluorescence densitometry at 546 nm (TLC Scanner 3, Camag Scientific Inc., Wilmington, NC).

### 4.3. DNA Extraction and PCR Amplifications

Fungal isolates were grown and DNA extraction was performed as described previously [66]. DNA concentration was adjusted to 5 ng/µl for PCR reactions. Partial gene fragments of β-tubulin (*benA*) (0.9 kb), calmodulin (*cmdA*) (1.2 kb), and nitrate reductase (*niaD*) (2.1 kb) genes were amplified (Table 5). The primer pair Bt3a-3b was designed in the current study to include the 5′ untranslated region of β-tubulin. Primers were designed based on genome sequence of *A. nomius* NRRL 13137 (GenBank accession no. JNOM01000216) using Primer3 version 0.4.0 [67,68]. PCR reactions were performed in 20 µl using 5 ng genomic DNA with a PCR premix (AccuPower^®^ HotStart, Bioneer, Alameda, CA, USA) on a MyCycler thermocycler (Bio-Rad Laboratories, Richmond, CA, USA) under the following conditions: for β-tubulin, 5 min at 94 °C followed by 35 cycles of 96 °C for 30 s, locus-specific annealing temperature for 1 min (Table 5), 72 °C for 1 min, and 5 min at 72 °C; for *cmdA* and *niaD* genes, 5 min at 94 °C followed by 38 cycles of 94 °C for 20 s, locus-specific annealing temperature for 30 s (Table 5), 72 °C for 1 min, and 5 min at 72 °C. Amplicons were visualized with SYBR Gold after 1.0% agarose gel electrophoresis and sequenced at the University of Arizona Genetics Core facility (UAGC, Tucson, AZ, USA) using primers mentioned in Table 5. For mating type analyses, portions of *MAT1-1* and *MAT1-2* were amplified and characterized from all 11 *A. texensis* isolates following [69] with slight modifications: 2 min at 95 °C followed by 30 cycles of 94 °C for 30 s, 54 °C for 30 s, 72 °C for 45 s, and 2 min at 72 °C. Amplicons were visualized using a 1.5% agarose gel. DNA sequences of representative isolates are deposited at Genbank (Table S1).

### 4.4. Molecular Analysis and Phylogenetics

Bidirectional sequences were used to create a consensus sequence of each amplicon. Gene segments were assembled with either two (*benA* and *cmdA*) or six (*niaD*) amplicons per gene, corrected manually and gene alignments were generated using the MUSCLE algorithm within Geneious Pro Version 7.1.9 (Biomatters Ltd., Auckland, New Zealand). Phylogenetic trees were generated for concatenated and individual gene sequences following Bayesian inference using MrBayes version 3.2.6 [70] and maximum likelihood (ML) analysis with PhyML at Phylogeny.fr [71,72] to confirm tree topologies. Bayesian inference was conducted by running Markov Chain Monte Carlo analysis for up to 10 million generations. For ML analysis, datasets were bootstrapped with 500 replicates. Trees were mid-point rooted using FigTree v.1.4.3 [73]. The presence of a complete *norB*-*cypA* sequence in the *A. texensis* aflatoxin biosynthesis gene cluster was confirmed with previously described primer sets [51].

### 4.5. Data Analysis

Total aflatoxin was measured in µg/g. Aflatoxin concentrations produced by each isolate and each taxon were analyzed using Analysis of Variance as implemented in JMP 11.1.1 (SAS Institute, Cary, NC, USA, 2013). Means were separated using Tukey’s HSD test (*p* = 0.05). Fungal colony diameters were measured in millimeters (mm). Diameters for each medium and temperature were compared among species with Tukey’s HSD (*p* = 0.05). Data were tested for normality prior to statistical analyses and, if required, log-transformed. True means are presented for clarity.

## Figures and Tables

**Table 1:** Origin of fungal isolates examined in the current study.

Isolate ^#^	Species/Taxon	Source	Citation
NRRL 13137	*A. nomius*	Wheat, USA	[37]
NRRL 443	*A. pseudotamarii*	Argentina	[36]
NRRL 25528	*A. caelatus*	Soil, USA	[38]
NRRL 465	*A. parasiticus*	USA	[39]
NRRL 29538	*A. parasiticus*	Soil, Georgia, USA	Horn B.W., National Peanut Lab, Dawson, GA (in NRRL database)
NRRL 29590	*A. parasiticus*	Soil, Georgia, USA	Horn B.W., National Peanut Lab, Dawson, GA (in NRRL database)
NRRL 424	*A. parasiticus*	Soil, Georgia, USA	Scales F.M. (in NRRL database)
NRRL 2999	*A. parasiticus*	Peanut, Uganda	[40]
BN009-E	*A. parasiticus*	Soil, Benin	None
NRRL A-11611	*A. minisclerotigenes*	Soil, Nigeria	[41,42]
4-2	*A. minisclerotigenes*	Soil, Australia	[43]
TAR3N43	*A. minisclerotigenes*	Peanut, Argentina	[16]
AF70/ATCC MYA-384	*A. flavus* S Morphotype	Soil, Arizona, USA	[32]
AF42/ATCC MYA-383	*A. flavus* S Morphotype	Soil, Arizona, USA	[32]
AF12/ATCC MYA-382	*A. flavus* S Morphotype	Soil, Arizona, USA	[32]
NRRL 3251	*A. flavus* S Morphotype	Walnut, California, USA	[41]
AF13/ATCC 96044	*A. flavus* L Morphotype	Soil, Arizona, USA	[32]
CHL019	*A. flavus* L Morphotype	Chili	[12]
CHL159	*A. flavus* L Morphotype	Chili	[12]
CHL187	*A. flavus* L Morphotype	Chili, Pakistan	[12]
W11P1R3-B R/NRRL 66855/A2292	*A. texensis*	Soil, Texas, USA	Current Study
EC42-I/NRRL 66856/A2295	*A. texensis*	Maize, Texas, USA	Current Study
BRG3224-E/NRRL 66857/A2296	*A. texensis*	Maize, Louisiana, USA	Current Study
WXMX1-1G/NRRL 66858/A2294	*A. texensis*	Soil, Texas, USA	Current Study
VCSSBC1-I/NRRL 66859/A2293	*A. texensis*	Maize, Arkansas, USA	Current Study
J35-E	*A. texensis*	Soil, Texas, USA	Current Study
VC16-A	*A. texensis*	Maize, Texas, USA	Current Study
CTL-1I	*A. texensis*	Soil, Texas, USA	Current Study
P2R2-A Q	*A. texensis*	Soil, Texas, USA	Current Study
1-1-O	*A. texensis*	Soil, Texas, USA	Current Study
1-1L	*A. texensis*	Soil, Texas, USA	Current Study
K805-E/A1170	Lethal Aflatoxicosis Fungus	Maize, Kenya	[44]
K784-D/A1168	Lethal Aflatoxicosis Fungus	Maize, Kenya	[44]
NRRL A-11612	*A. aflatoxiformans*	Groundnut, Nigeria	[17,41]
BN040-B/ATCC MYA-381	*A. aflatoxiformans*	Soil, Benin	[17,45]
BN038-G/ATCC MYA-380	*A. aflatoxiformans*	Soil, Benin	[17,45]
BN008-R/ATCC MYA-379	*A. aflatoxiformans*	Soil, Benin	[17,45]

^#^ Isolates were obtained from the ARS Culture Collection, Peoria, IL, USA (NRRL), the American Type Culture Collection, Manassas, USA (ATCC), Fungal Genetics Stock Center, Manhattan, KS (A) or were present at the USDA-ARS, Tucson Laboratory Culture Collection.

**Table 2:** Production of major mycotoxins ^a^ by certain species within *Aspergillus* section *Flavi*.

Species	Aflatoxin B_1_	Aflatoxin B_2_	Aflatoxin G_1_	Aflatoxin G_2_	CPA	Aspergillic Acid
*A. texensis*	+	+	+	+	+	+
*A. flavus*	+	+	−	−	±	+
*A. minisclerotigenes*	+	+	+	+	+	+
*A. parasiticus*	+	+	+	+	−	+

^a^ Data from [35,42] and the current study.

**Table 3:** Aflatoxin production by fungi with S morphology within *Aspergillus* section *Flavi*.

Species	Isolate	Source/Location	Aflatoxin (µg/g)		
			AFB	AFG	Total AF
*A. texensis*	BRG3224 E	Maize/USA	42	111	153
	EC42-I	Maize/USA	40	99	139
	W11P1R3-B R	Soil/USA	47	111	158
	**Average**		**43C**	**107B**	**150B**
*A. flavus* S Morphotype	AF12	Soil/USA	190	NA	190
	AF42	Soil/USA	181	NA	181
	AF70	Soil/USA	191	NA	191
	**Average**		**187AB**	NA*	**187B**
*A. minisclerotigenes*	A-11611	Groundnut/Nigeria	31x	72x	103x
	4-2	Soil/Australia	9y	24y	33y
	TAR3N43	Soil/Argentina	9y	25y	34y
	**Average**		**17D**	**40C**	**57C**
*A. aflatoxiformans*	A-11612	Groundnut/Nigeria	92y	202y	294y
	BN038-G	Soil/Benin	105y	129z	234y
	BN040-B	Soil/Benin	207x	361x	568x
	**Average**		**134B**	**231A**	**365A**
Lethal Aflatoxicosis Fungus	K805-E	Maize/Kenya	304	NA	304xy
	K784-D	Maize/Kenya	361	NA	361x
	K849-B	Maize/Kenya	215	NA	215y
	**Average**		**294A**	NA*	**294A**

B aflatoxin, G aflatoxin and total aflatoxin concentrations were compared between and within species. Each toxin concentration is a mean of four replicates. Means followed by different upper case letters in bold (**A,B,C**) or lower case letters (x,y) within a column differ significantly according to Tukey’s HSD (*p* < 0.01). Values within a column lacking a letter do not differ (ANOVA, *p* > 0.05). * Fungi which do not produce G aflatoxins were excluded when comparing G aflatoxin production between and within species.

**Table 4:** Influence of temperature on radial growth of three S morphology species in *Aspergillus* section *Flavi* on agar media.

Medium ^Φ^	Species	Colony Diameter (mm) ^ρ^							
		15 °C	20 °C	25 °C	30 °C	35 °C	37 °C	40 °C	42 °C
CZ	*A. texensis*	3^b^	31^b^	53^ab^	67	73	55^b^	46	7
	*A. flavus*	7^a^	35^b^	50^b^	66	65	54^b^	37	8
	*A. minisclerotigenes*	3^b^	41^a^	62^a^	75	75	63^a^	39	8
									
CZ with NaCl	*A. texensis*	11	44	60	72	77	72	68	13
	*A. flavus*	12	44	57	69	71	65	69	12
	*A. minisclerotigenes*	10	44	66	73	77	72	73	15
									
CYA	*A. texensis*	9	46	71	77	78	65	54	9
	*A. flavus*	11	42	64	72	71	57	56	11
	*A. minisclerotigenes*	9	44	68	77	78	64	49	10
									
Malt	*A. texensis*	7^a^	30	46^ab^	68	69	55	41	11
	*A. flavus*	5^ab^	34	44^b^	57	67	55	30	7
	*A. minisclerotigenes*	3^b^	43	69^a^	67	68	58	32	8
									
V8	*A. texensis*	4^a^	33	60	68	72	52^b^	42^a^	7
	*A. flavus*	3^ab^	32	53	66	68	61^a^	28^b^	7
	*A. minisclerotigenes*	2^b^	31	59	74	73	67^a^	31^b^	9

^Φ^ Media compositions in Materials and Methods; CZ-Czapek solution agar, CZ with NaCl-Czapek solution agar with 2% NaCl, CYA-Czapek solution with yeast extract agar, Malt-Malt solution agar, V8-V8 juice agar. ^ρ^ Colony diameter 7 d post inoculation. Statistical comparisons among species were performed independently for each medium/temperature combination. Values are means of four replicates; means followed by the same letter do not differ significantly (Tukey’s HSD, *p* > 0.05).

**Table 5:** Primers and locus specific annealing temperatures (Ta) for PCR amplifications.

Primer Pair	Target Gene	Sequence	T_a_ (°C)	Reference
Bt2a-2b	β-tubulin	F-GGTAACCAAATCCGTGCTGCTTTC	51	[50]
		R-ACCCTCAGTGTAGTGACCCTTGGC		
Bt3a-3b		F-CGTCGTTCATTCGAGGTGTA	56	Current study
		R-CCGCTCAACTTCAAGTCCAT		
cmd42-637	Calmodulin	F-GGCCTTCTCCCTATTCGTAA	56	[16]
		R-CTCGCGGATCATCTCATC		
cmd2F-2R		F-GGCTGGATGTGTGTAAATC	48	[16]
		R-ATTGGTCGCATTTGAAGGG		
niaDF-AR	Nitrate reductase	F-CGGACGATAAGCAACAACAC	52	[16]
		R-GGATGAACACCCGTTAATCTGA		
niaDBF-BR		F-ACGGCCGACAGAAGTGCTGA	57	[16]
		R-TGGGCGAAGAGACTCCCCGT		
niaDCF-CR		F-GCAGCCCAATGGTCACTACGGC	55	[12]
		R-GGCTGCACGCCCAATGCTTC		

## Supplementary Materials

The following are available online at http://www.mdpi.com/2072-6651/10/12/513/s1, Figure S1: Mid-point rooted Bayesian phylogeny of *A. texensis* and closely related S morphology fungi with several additional species for reference based on partial calmodulin gene, *cmdA* (1.2 kb). Values above nodes are Bayesian posterior probabilities and values below nodes are bootstrap values from 500 replicates. Figure S2: Mid-point rooted Bayesian phylogeny of *A. texensis* and closely related S morphology fungi with several additional species for reference based on a portion of nitrate reductase gene, *niaD* (2.1 kb). Values above nodes or before commas are Bayesian posterior probabilities and values below nodes or after commas are bootstrap values from 500 replicates. Table S1: Isolates used in the current study with GenBank accession numbers. Sequences recovered from GenBank are indicated in bold.
