# Supplementary material for: Aspergillus texensis: A Novel Aflatoxin Producer with S Morphology from the United States
Source: Toxins (Basel). 2018 Dec 3;10(12):513. doi: 10.3390/toxins10120513 (PMC6316697; doi:10.3390/toxins10120513)
Supplement: Supplementary file 1 [file toxins-10-00513-s001.pdf]

Supplementary Materials: *Aspergillus texensis*: A novel aflatoxin producer with S morphology from the United States

Pummi Singh, Marc J. Orbach and Peter J. Cotty

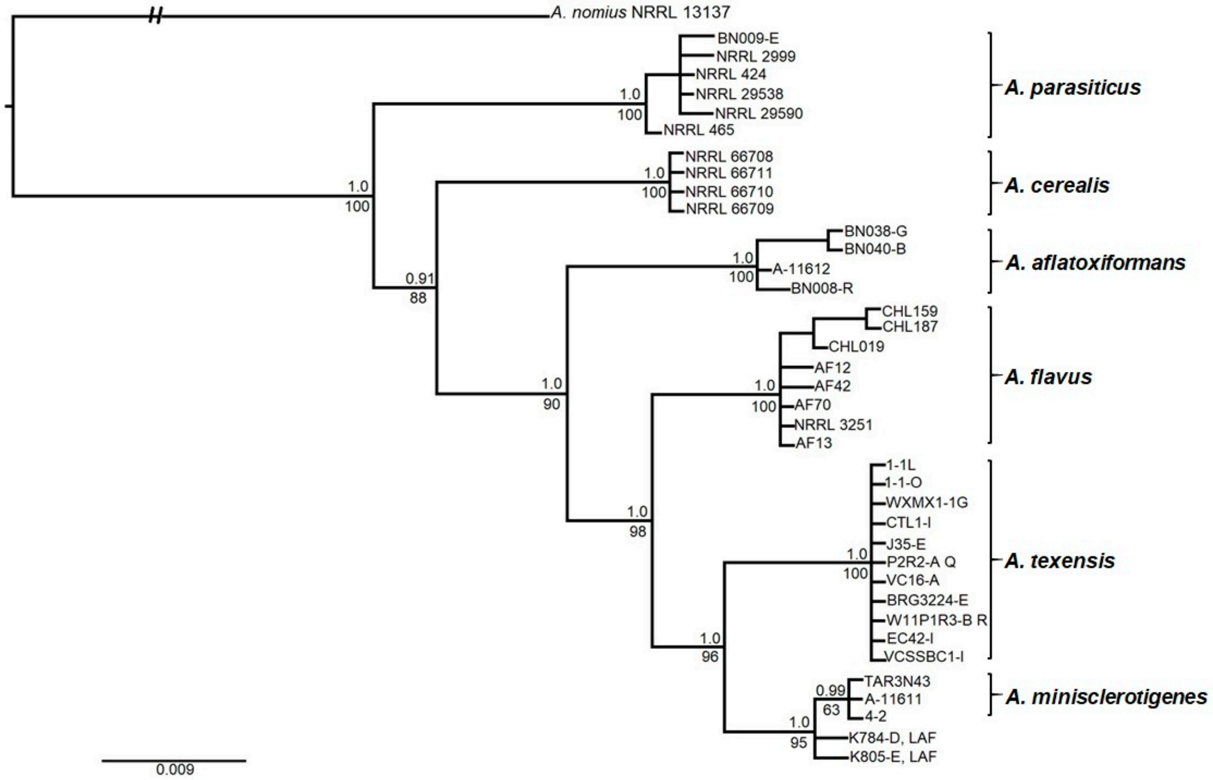

Figure S1. Mid-point rooted Bayesian phylogeny of *A. texensis* and closely related S morphology fungi with several additional species for reference based on partial calmodulin gene, *cmdA* (1.2 kb). Values above nodes are Bayesian posterior probabilities and values below nodes are bootstrap values from 500 replicates.

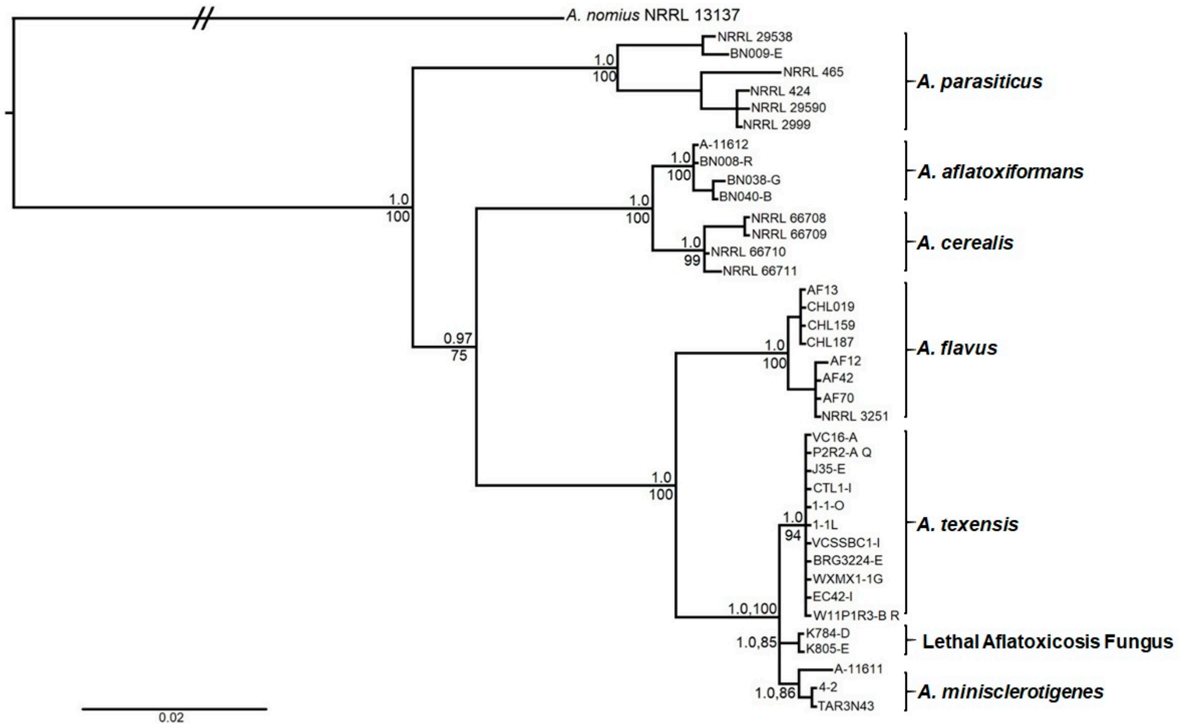

Figure S2. Mid-point rooted Bayesian phylogeny of *A. texensis* and closely related S morphology fungi with several additional species for reference based on a portion of nitrate reductase gene, *niaD* (2.1 kb). Values above nodes or before commas are Bayesian posterior probabilities and values below nodes or after commas are bootstrap values from 500 replicates.

Table S1. Isolates used in the current study with GenBank accession numbers. Sequences recovered from GenBank are indicated in bold.

Species	Isolate	GenBank Accession Number		
		benA	***cmdA***	***niaD***
Aspergillus nomius	NRRL 13137	MK119726	MK119692	**MH760519**
Aspergillus minisclerotigenes	TAR3N43	MK119727	MK119693	**MH760520**
Aspergillus minisclerotigenes	4-2	MK119728	MK119694	**MH760522**
Aspergillus minisclerotigenes	NRRL A-11611	MK119729	MK119695	**MH760525**
Aspergillus flavus	CHL159	MK119730	MK119696	**MH760528**
Aspergillus flavus	CHL187	MK119731	MK119697	**MH760529**
Aspergillus flavus	AF13	MK119732	MK119698	**MH760530**
Aspergillus flavus	AF12	MK119733	MK119699	**MH760531**
Aspergillus flavus	AF42	MK119734	MK119700	**MH760532**
Aspergillus flavus	AF70	MK119735	MK119701	**MH760533**
Aspergillus flavus	NRRL 3251	MK119736	MK119702	**MH760534**
Aspergillus parasiticus	NRRL 2999	MK119737	MK119703	**MH760537**
Aspergillus parasiticus	BN009-E	MK119738	MK119704	**MH760538**
Aspergillus parasiticus	NRRL 424	MK119739	MK119705	MK119671
Aspergillus parasiticus	NRRL 465	MK119740	MK119706	MK119672
Aspergillus parasiticus	NRRL 29538	MK119741	MK119707	MK119673
Aspergillus parasiticus	NRRL 29590	MK119742	MK119708	MK119674

Aspergillus cerealis	NRRL 66708	MK119743	MK119709	MK119675
Aspergillus cerealis	NRRL 66709	MK119744	MK119710	MK119676
Aspergillus cerealis	NRRL 66710	MK119745	MK119711	MK119677
Aspergillus aflatoxiformans	NRRL A-11612	MK119746	MK119712	MK119678
Aspergillus aflatoxiformans	BN038-G	MK119747	MK119713	MK119679
Aspergillus aflatoxiformans	BN040-B	MK119748	MK119714	MK119680
Aspergillus aflatoxiformans	BN008-R	MK119749	MK119715	MK119681
Unnamed lineage Lethal Aflatoxicosis Fungus	A1168	MK119750	MK119716	MK119682
Unnamed lineage Lethal Aflatoxicosis Fungus	A1170	MK119751	MK119717	MK119683
Aspergillus texensis	NRRL 66855	MK119752	MK119718	MK119684
Aspergillus texensis	NRRL 66856	MK119753	MK119719	MK119685
Aspergillus texensis	NRRL 66857	MK119754	MK119720	MK119686
Aspergillus texensis	NRRL 66858	MK119755	MK119721	MK119687
Aspergillus texensis	NRRL 66859	MK119756	MK119722	MK119688
Aspergillus pseudotamarii	NRRL 443	MK119757	MK119723	MK119689
Aspergillus tamarii	NRRL 20818	MK119758	MK119724	MK119690
Aspergillus caelatus	NRRL 25528	MK119759	MK119725	MK119691
